# Anti-*Trypanosoma cruzi* Effect of Fatty Acids from *Porcelia macrocarpa* Is Related to Interactions of Cell Membranes at Different Microdomains as Assessed Using Langmuir Monolayers

**DOI:** 10.1021/acsomega.5c01382

**Published:** 2025-05-21

**Authors:** Ivanildo A. Brito, Matheus E. Rosa, Elodie Boisselier, Vanessa Albuquerque, Andre G. Tempone, Luciano Caseli, João Henrique G. Lago

**Affiliations:** † Center of Natural and Human Sciences, 74362 Federal University of ABC, 09210-170 Santo Andre, SP, Brazil; ‡ Department of Chemistry, Federal University of São Paulo, 09972-270 Diadema, SP, Brazil; § Faculty of Medicine, Université Laval, Quebec City G1 V 0A6, Quebec, Canada; ∥ Physiopathology Laboratory, 196591 Butantan Institute, 05503-900 São Paulo, SP, Brazil

## Abstract

Chagas disease, a parasitic disease caused by the protozoan Trypanosoma cruzi, is an important health problem affecting more than 8 million people worldwide. The only available treatments, benznidazole and nifurtimox, display high toxicity and reduced efficacy in the chronic phase of the disease. To find new natural products with anti-T. cruzi activity, the CH_2_Cl_2_ extract of Porcelia macrocarpa R. E. Fries (Annonaceae) seeds was subjected to bioactivity-guided fractionation. Through several chromatographic steps, one group consisting of a mixture of 10 chemically related fatty acids (**1**–**10**) was obtained. This group showed activity against trypomastigote forms with an EC_50_ of 4.0 μg/mL, similar to the standard drug benznidazole (EC_50_ = 3.9 μg/mL). It also showed activity against the intracellular amastigotes, with an EC_50_ of 0.5 μg/mL, close to the efficacy of benznidazole (EC_50_ = 0.9 μg/mL). In addition, the mixture of **1**–**10** showed no toxicity against murine fibroblasts (CC_50_ > 200 μg/mL), resulting in SI > 49 and >416 in trypomastigotes and amastigotes, respectively. The interaction of the mixture with the protozoan membrane models was also assessed with Langmuir monolayers composed of three phosphatidylethanolamine (PE) lipids with different degrees of acyl chain unsaturation and in the presence of mucins. Compounds **1–10** favorably interact with all tested lipids, with maximum insertion pressure (MIP) values above 40 mN/m and positive synergy values, suggesting penetration through the mucins. Furthermore, the mixture has a higher affinity for monounsaturated lipids bound to mucins, with an MIP value of 57.59 ± 2.59 mN/m. Based on these results, the effect of compounds **1**–**10** against T. cruzi can be related to interactions with the parasite cell membranes.

## Graphical abstract

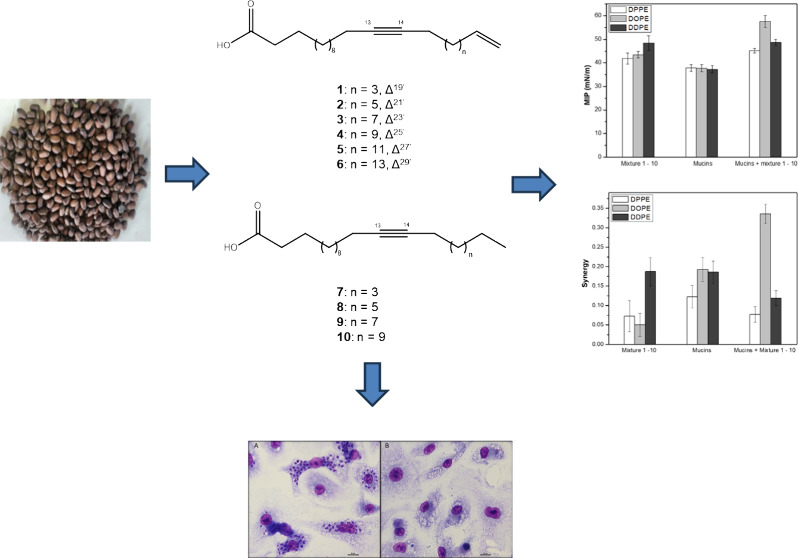

## 1 Introduction

Chagas disease, caused by the protozoan Trypanosoma cruzi, is one of the most relevant Neglected Tropical Diseases for the WHO. Approximately 8 million people are infected, in addition to 30,000 new cases and 10,000 deaths per year due to clinical complications of the disease. It is estimated that about 75 million people are at risk of contracting the disease.^1^ Current treatment options are limited to only two drugs: benznidazole and nifurtimox, both of which have limited efficacy and are associated with intense side effects.^2,3^ In this scenario, natural products are important sources of anti-T. cruzi compounds.

Porcelia macrocarpa (Warm.) R. E. Fries (Annonaceae) produces different metabolites with antiparasitic activity, especially acetylenic acetogenins^4–6^ and fatty acids.^7,8^ As reported,^4,8^ the anti-T. cruzi effect of these compounds is associated, at least in part, with the alteration of the electrical potential of the plasma membrane and/or the depolarisation of the plasma membranes, thereby disrupting the bioenergetic system. As part of our ongoing studies with this plant species, the present work describes the obtaining of a mixture of 10 biosynthetic-related acetylenic fatty acids (**1–10**), obtained through bioactivity-guided fractionation. Compounds **1–10** were chemically identified by NMR and UHPLC/ESI-HRMS analysis, and this mixture was submitted for evaluation of its efficacy against trypomastigote (extracellular) and amastigote (intracellular) forms of T. cruzi. To gather molecular-level information about the interaction between the active mixture and the membrane of these microorganisms, it is helpful to use models that allow for the modulation of membrane properties and the investigation of binding parameters between lipidic interfaces and biologically relevant compounds. Langmuir monolayers are a powerful tool for mimicking the inner and outer leaflets of cell membranes, enabling easy modulation of properties such as lipid and buffer composition, physical state, and molecular density, as described in the literature.^9–12^ In this study, Langmuir films with phospholipids bearing phosphatidylethanolamine (PE) as polar heads were employed since they are the major lipid constituents of the T. cruzi cell membrane.^13^ Additionally, mucins, which are highly glycosylated proteins, cover the cell membrane of T. cruzi and other protozoa, playing important roles such as the protection of the parasite and adhesion to host cells.^14^ Models of the T. cruzi membrane were thus employed by injecting a mixture of mucins into the subphase of different Langmuir monolayers composed of various PE phospholipids to assess their influence on the membrane binding of the active compounds.

## 2 Results and Discussion

### 2.1 Chemical Characterization of **1**–**10**

The chemical characterization of compounds **1**–**10** was performed by the analysis of NMR and UHPLC/ESI-HRMS data. Initially, the ^1^H NMR spectrum of the mixture of **1**–**10** showed characteristic signals of a terminal double bond in a side chain at δ_H_ 5.80 (dt, *J* = 16.9, 10.2, and 6.6 Hz) and at δ_H_ 4.96 (m). Additionally, the presence of a terminal methyl of the side chain was also suggested by the observation of a triplet at δ_H_ 0.87 (*J* = 6.9 Hz). Furthermore, this spectrum showed one broad singlet at δ_H_ 1.25 and one triplet at δ_H_ 2.34 (*J* = 7.4 Hz, H-2) corresponding to hydrogens of the methylene side chain characteristic of fatty acids.^7^ The presence of one triplet at δ_H_ 2.24 (*J* = 7.0 Hz, H-12 and H-15, respectively), compatible with propargyl hydrogens, suggested the presence of a triple bond in the side chain, as previously observed in acetogenins isolated from P. macrocarpa.^6^ ^13^C NMR and DEPT spectra displayed signals referring to sp^2^ carbons at δ_C_ 139.2 (CH) and 114.3 (CH_2_), confirming the presence of the terminal double bond, in addition to a signal at δ_C_ 14.2 (CH_3_) attributed to the terminal methyl group. It was also possible to observe the presence of two signals referring to sp carbons at δ_C_ 80.4 and 80.3 (C-13′ and C-14′) and their adjacent methylene carbons at δ_C_ 18.8 (C-12′) and 18.8 (C-15′). Finally, the signal at δ_C_ 173.7 was assigned to the carboxylic carbon (C-1). Based on these data, a mixture of acetylene fatty acids was proposed.

Analysis by UHPLC/ESI-HRMS indicated the predominance of 10 compounds with molecular formulas C_20_H_34_O_2_ (**1**), C_22_H_38_O_2_ (**2**), C_24_H_42_O_2_ (**3**), C_26_H_46_O_2_ (**4**), C_28_H_50_O_2_ (**5**), C_30_H_54_O_2_ (**6**), C_20_H_36_O_2_ (**7**), C_22_H_40_O_2_ (**8**), C_24_H_44_O_2_ (**9**), and C_26_H_48_O_2_ (**10**), which were established due to the presence of the [M–H]^−^ ion peaks at *m*/*z* 305.2479, 333.2790, 361.3103, 389.3417, 417.3730, 445.4043, 307.2634, 335.2948, 363.3260, and 391.3574, respectively. Finally, the positioning of the triple bond in the side chain for compounds **1**–**10** was determined at C-13′ and 14′ through MS/MS analysis, which showed a signal at *m*/*z* 249, referring to the fragmentation at C-16′ to form a 15′-en-13′-yn conjugated system, as illustrated in Figure 1.

**Figure 1:**
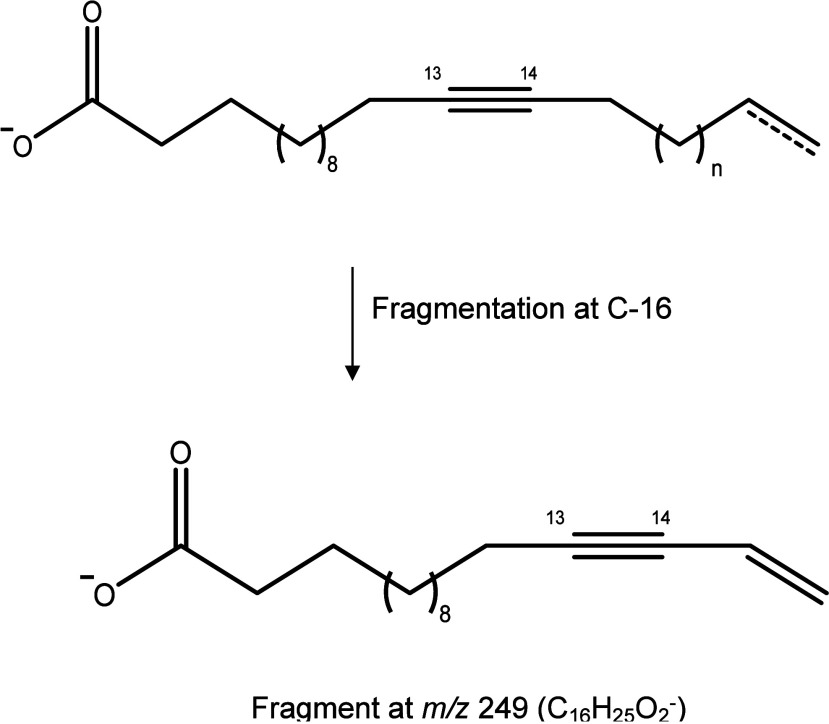
Proposed fragmentation in MS/MS spectra (negative mode) of **1**–**10**.

Therefore, through a comparative analysis of the spectral data obtained with the existing records in the literature,^7,8^ the presence of a mixture of 10 biosynthetically related fatty acids, such as icos-19-en-13-inoic acid (**1**), docos-21-en-13-inoic acid (**2**), tetracos-23-en-13-inoic acid (**3**), hexacos-25-en-13-inoic acid (**4**), octacos-27-en-13-inoic acid (**5**), triacont-29-en-13-inoic acid (**6**), icos-13-inoic acid (**7**), docos-13-inoic acid (**8**), tetracos-13-inoic acid (**9**), and hexacos-13-inoic acid (**10**) is observed, as indicated in Figure 2; this is the first report on compounds **1** and **3**–**10** in the literature.

**Figure 2:**
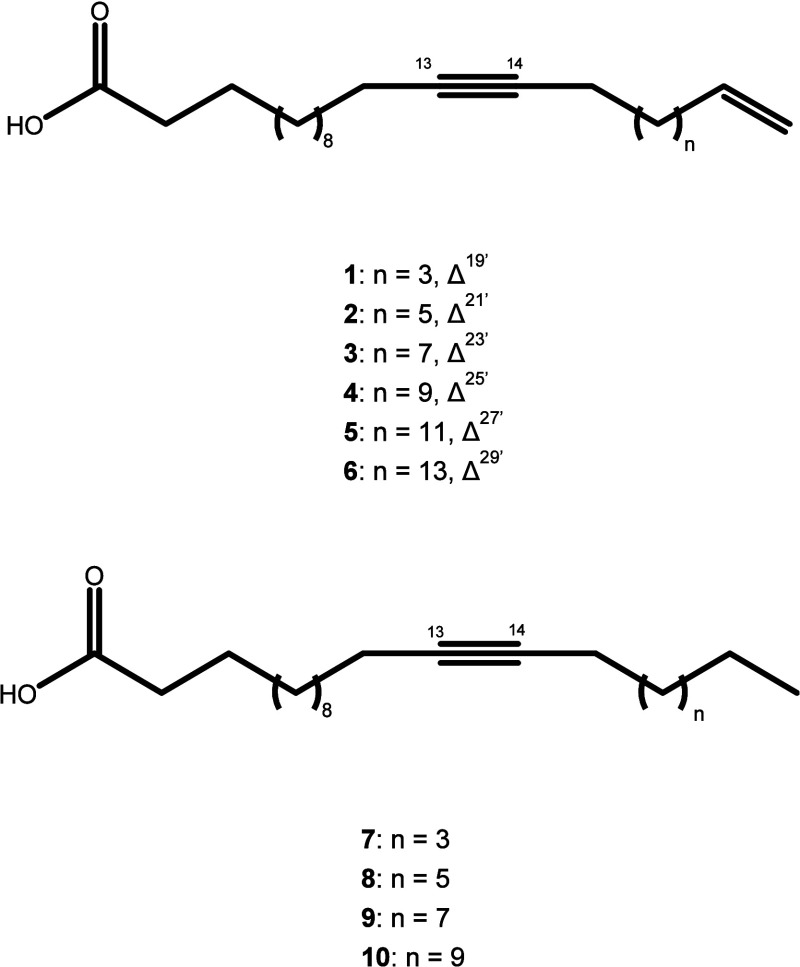
Structures of fatty acids **1**–**10** identified in P. macrocarpa seeds.

### 2.2 Bioactivity of **1**–**10** against *T. cruzi*

The anti-T. cruzi activity of the mixture of **1**–**10** was evaluated against trypomastigote (extracellular) and amastigote (intracellular) forms, whereas the cytotoxicity was determined against NCTC mammalian cells. The obtained results (Table 1) demonstrate that the mixture of **1**–**10** induced no cytotoxicity to NCTC cells at the highest tested concentration (CC_50_ > 200 μg/mL).

**Table 1:** EC_50_ Values against Trypomastigotes (TRYPO) (24 h) and Amastigotes (AMA) (48 h) Forms of *T. cruzi*, CC_50_ in Mammalian Cells (NCTC) and SI of the Mixture of **1**–**10** ^a^

compound	*T. cruzi* EC_50_ (μg/mL)		NCTC CC_50_ (μg/mL)	SI	
	TRYPO	AMA		TRYPO	AMA
**1**–**10**	4.0 ± 1.1	0.5 ± 0.1	>200	>49.3	>416.6
benznidazole	3.9 ± 1.2	0.9 ± 0.2	>200	>51.2	>210.5

a EC_50_: 50% Effective concentration; CC_50_: 50% cytotoxic concentration (NCTC cells); SI: selectivity index; benznidazole: standard drug.

According to the nonlinear regression curves of mixture of **1–10** against amastigotes and trypomastigotes, 100% of the parasites were eliminated at the highest concentrations (Figure 3).

**Figure 3:**
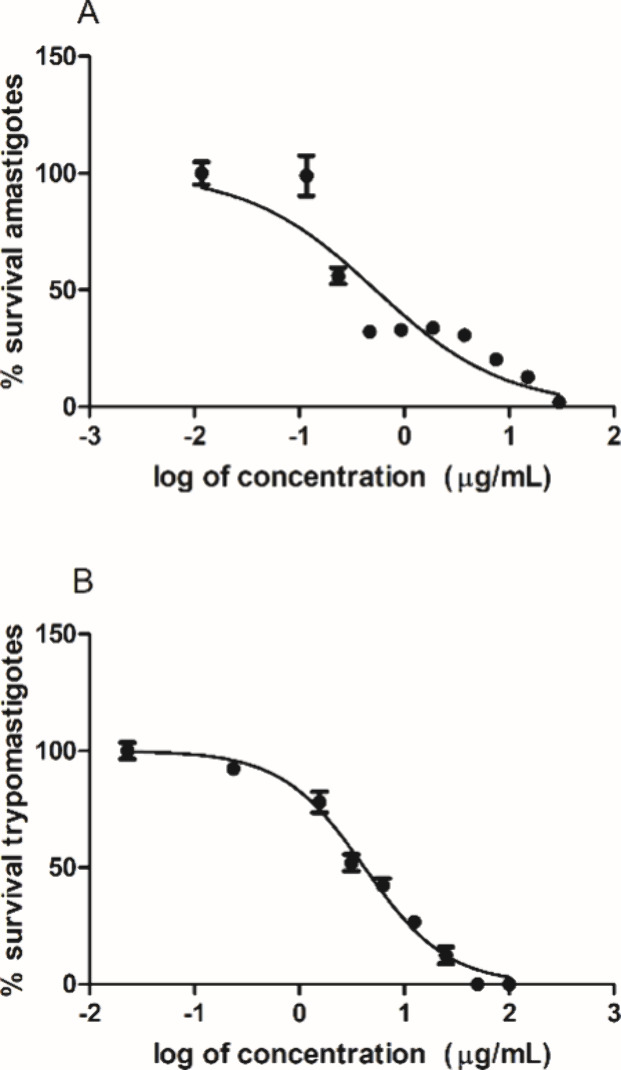
Nonlinear regression curves of mixture of **1**–**10** obtained after treatment of intracellular amastigotes (A) (48 h) and trypomastigotes (B) (24 h). Curves were obtained using GraphPad Prism software.

When tested against trypomastigotes of T. cruzi, the mixture of **1**–**10** showed an EC_50_ value of 4.0 ± 1.1 μg/mL, which resulted in a selectivity index (SI) higher than 49. Benznidazole showed an EC_50_ value of 3.9 ± 1.2 μg/mL and an SI > 51. When tested against the amastigote forms, the mixture of **1**–**10** was effective with an EC_50_ value of 0.5 ± 0.1 μg/mL and an SI higher than 400. Benznidazole showed an EC_50_ value of 0.9 ± 0.2 μg/mL and an SI > 210. As demonstrated in Figure 4, the mixture of **1**–**10** eliminated >90% of the intracellular amastigotes, without affecting the morphology of the host mammalian cells at the tested concentration. The infection ratio of macrophages (untreated control) was 55% after 48 h of incubation.

**Figure 4:**
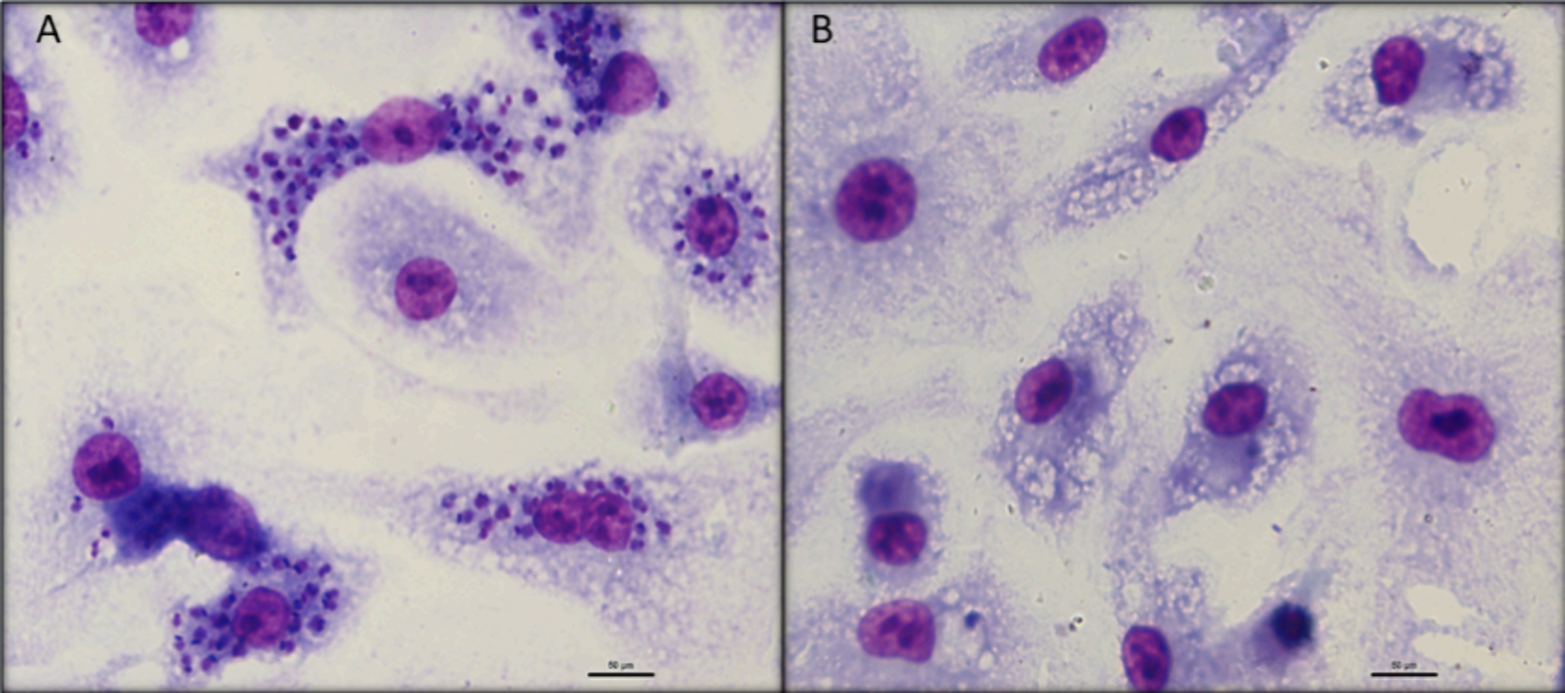
Micrographs of T. cruzi-infected macrophages. (A) Untreated macrophages. (B) Macrophages after treatment with a mixture of **1**–**10** (30 μg/mL) for 48 h. Magnification 100×. Bar represent 50 μm.

The comparative analysis of the results with the data for stearic acid, a fully saturated fatty acid that showed inactivity against T. cruzi,^4^ suggests that the presence of the triple bond emerges as a crucial element for biological activity. It is also important to note that 9-octadecinoic acid has been reported in the evaluation of antiparasitic activity, showing an inhibitory capacity in the growth of T. cruzi epimastigotes, suggesting that the presence of the triple bond in the side chain is important for biological activity.^15^ Another relevant structural aspect is the finding that the presence of conjugated triple bonds in the structure resulted in a cytotoxic effect on mammalian cells, as reported for macrocarpathic acid (12,14-octadecadiinoic acid).^16^ In contrast, no cytotoxic effect was observed for the mixture of **1**–**10**, which has no conjugated triple bonds. Therefore, these data suggest that the absence of conjugated triple bonds may be responsible for the reduced cytotoxicity for NCTC cells.

Acetylenic compounds have been shown to be an important class of substances of pharmacological interest, especially antiparasitic activity. Previous studies with octadec-9-inoic acid, isolated from the flowers of P. macrocarpa, showed effective results against trypomastigote forms of T. cruzi. This finding suggests that the mechanism of action is related, at least in part, to the alteration of the electrical potential of the plasma membrane (Δψ*p*).^8^ In addition, previous studies report that compound **2** (docos-21-en-13-inoic acid) displayed in vitro activity against amastigote forms of L. (L.) infantum *.* ^7^

### 2.3 Binding of **1**–**10** to Membrane Models

A useful tool to gather information about the interaction of proteins and bioactive compounds from membranes is the measurement of their maximum insertion pressure (MIP). This method, widely utilized in the literature,^17^ corresponds to the surface pressure at which the compound no longer interacts with the monolayer. The binding parameters of the mixture of **1–10** were measured using membrane models with pure lipids (ranging from fully saturated to highly unsaturated acyl chains) as well as in the presence of mucins previously injected underneath these lipidic interfaces. DPPE is composed of two saturated acyl chains (diC_16:0_), whereas DOPE has two monounsaturated acyl chains (diC_18:0_), and DDPE has two polyunsaturated chains with six unsaturations each (diC_22:6_). These different unsaturation patterns lead to changes in their organization and two-dimensional physical states. Unsaturated acyl chains favor the liquid-expanded (LE) state, more disordered and with a larger area per molecule than the liquid-condensed (LC) and solid-condensed (SC), which are well-packed states of saturated acyl chains. The MIP values will be compared to the value of 30 mN/m, which is defined as the presumed physiological lateral pressure in real membranes.^18,19^ Regarding the mixture of **1–10**, the MIP values increase as the unsaturation on the lipid alkyl chains increases, with values of 41.9 ± 2.4, 43.4 ± 1.4, and 48.4 ± 3.0 mN/m for DPPE, DOPE, and DDPE, respectively (Figure 5). This trend indicates that the mixture of compounds is more likely to bind to fluid, less sterically hindered domains of the membranes.

**Figure 5:**
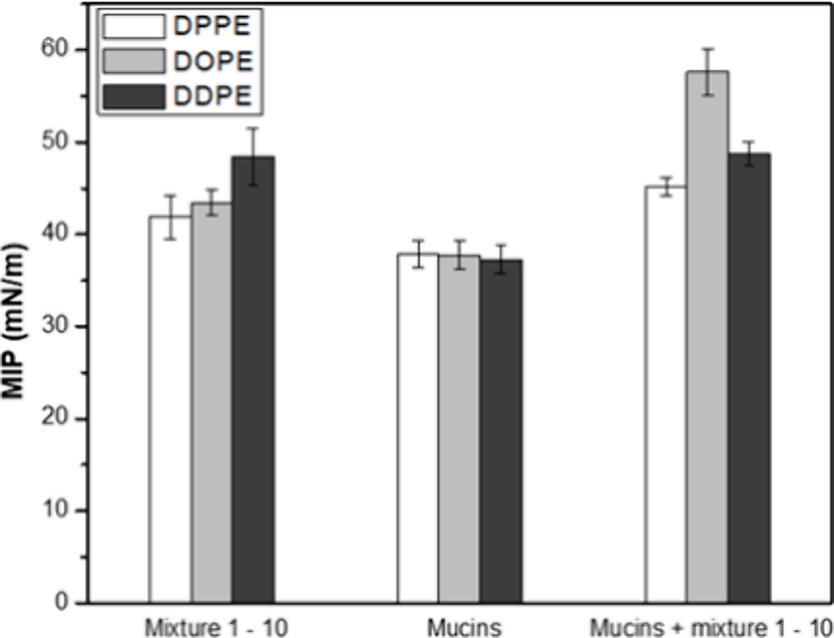
Bar plots for the maximum insertion pressure (MIP) of a mixture of **1–10**, mucins, and a mixture of **1–10** and mucins, in DPPE, DOPE, and DDPE monolayers.

Regarding the mucins, the MIP values are quite similar (37.8 ± 1.5, 37.8 ± 1.5, and 37.2 ± 1.5 mN/m for DPPE, DOPE and DDPE respectively), meaning that changes in the lipid alkyl chains have little impact on their binding and mucins are able to bind to those membranes at domains of different fluidities. The ternary system was then established by spreading the lipids (first surface pressure equilibrium), injecting the mucin lipids (second surface pressure equilibrium), and then injecting the mixture of **1–10** (third surface pressure equilibrium). To correctly address the effects of the injection of the mixture into the mucin + lipid model, the final MIP values must be compared to those of mucins in the presence of the different lipids. Since the MIP values of this model are considerably higher than for only mucins (45.1 ± 1.0, 57.6 ± 2.6, and 48.8 ± 12 mN/m for DPPE, DOPE, and DDPE, respectively), it can be stated that the mixture of compounds can penetrate through the mucin barrier and interact with the lipid monolayers. Moreover, the MIP value for the mixture of **1–10** with mucins in DOPE (57.59 ± 2.59 mN/m) is strikingly higher than the values observed with other lipids. This is possibly related to the least steric hindrance for the monounsaturated DO chains, which have higher molecular areas in comparison to saturated DP and polyunsaturated DD chains at the same pressure, as observable in pressure–area isotherms for DPPC, DOPE, and DDPE.^20,21^ The synergy is another parameter allowing to indicate attractive interactions (positive values of synergy) or repulsive interactions (negative values of synergy.^22^ All synergy values, represented in Figure 6, are positive, meaning that the interactions between the mixture, mucins, and lipids are mainly attractive.

**Figure 6:**
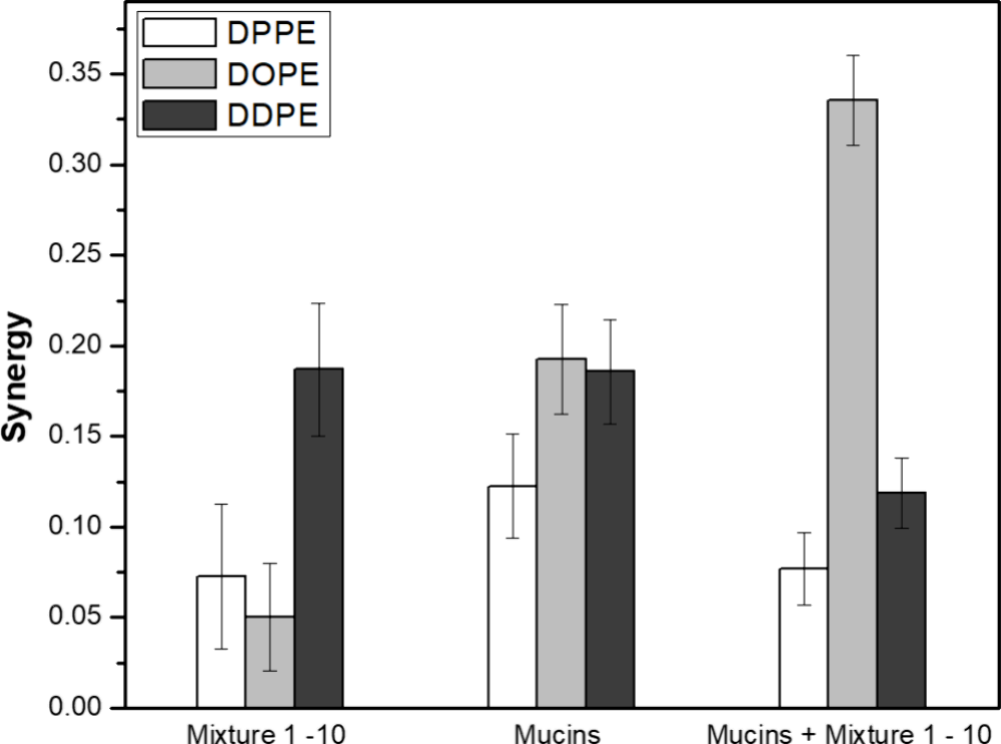
Bar plots for the synergy of mixture of **1–10**, mucins, and a mixture of **1–10** and mucins, in DPPE, DOPE, and DDPE monolayers.

Regarding the mucins alone, the synergy values are higher for unsaturated lipids (i.e., DOPE and DDPE) than for the saturated DPPE, even though MIP values in the three lipids are similar, as described before. It is possible that the higher molecular areas for the unsaturated acyl chains may better accommodate the mucins with less steric hindrance.

For the mixture of **1–10** in lipids, the synergy for DDPE (0.19 ± 0.04) was considerably higher than that for other lipids, meaning that the didocosahexaenoyl (DD) acyl chains have a packing that favors the binding of the compounds. It is possible, in this case, that the long DD chains favor hydrophobic interactions with the alkyl chains of compounds **1–10**. Regarding the mixture of compounds in the models with mucins and lipids, synergy with DOPE (0.34 ± 0.02) is significantly higher than all other values measured. The highest values of MIP and synergy were observed in the presence of DOPE, meaning that the dioleoyl (DO) chains have an optimal configuration for the insertion of the compounds in the presence of a mucoid layer. Given that chemically related acetogenin activity comes mostly from their mitochondrial NADH dehydrogenase inhibition,^23^ the drugs must be able to penetrate the parasite cell membrane to reach its intracellular organelles. As seen by the maximum insertion pressure measurements, the mixture of **1–10** is likely to penetrate the mucin layer and interact with the lipids in the cell membrane at different microdomains, which should promote their bioactivity.

As a result, the Langmuir monolayer results provided insights into the potential toxicity of the tested compounds by highlighting their ability to interact with specific membrane components. The high MIP values and strong synergy observed with DOPE suggest that the dioleoyl chains provide an optimal configuration for the insertion of the compounds, particularly in the presence of a mucoid layer. This indicates that the compounds can effectively penetrate the mucin barrier and integrate into the lipid microdomains of the cell membrane. Given that the bioactivity of chemically related acetogenins is attributed to their mitochondrial NADH dehydrogenase inhibition, the ability of these compounds to disrupt membrane organization and access intracellular targets is likely a key factor in their cytotoxic effects. These findings support the hypothesis that the tested compounds exhibit toxicity by leveraging specific lipid interactions to reach and impair essential intracellular organelles.

## 3 Conclusions

The bioactivity-guided phytochemical study conducted on the CH_2_Cl_2_ extract from P. macrocarpa seeds allowed for the characterization of 10 chemically related fatty acids (**1–10**), which were characterized by NMR and UHPLC/ESI-HRMS analysis. The mixture of **1**–**10** showed selective antitrypanosomal activity, eliminating both clinical forms of the parasite, the trypomastigotes and the intracellular amastigotes, showing no mammalian toxicity to murine fibroblasts. Regarding the interaction with the membrane environment, the mixture of **1–10** interacts favorably with saturated and unsaturated PE lipids, although it shows a preference for unsaturated and longer alkyl chains. These chemically related compounds can penetrate a mucin layer, with a higher affinity for DOPE in this scenario. Therefore, the results obtained in this study, together with previous findings, clearly demonstrate the effects of these acetylenic fatty acids are associated with the membrane of the parasite. This data could contribute to future studies in the development of new therapies for Chagas disease.

## 4 Materials and Methods

### 4.1 General

NMR spectra were recorded on a Varian INOVA 500 instrument using CDCl_3_ and TMS (both from Aldrich) as a solvent and internal standard, respectively. Silica gel 60 and silica gel 60 PF_254_ (Merck, Darmstadt, Germany) were used for the CC and TLC procedures, respectively. UHPLC separations were performed on an Ultimate 3000 system (Dionex, Sunnyvale, CA, USA) equipped with a quaternary pump system, a PDA detector, and a Phenomenex Luna reversed-phase C_18_ column (250.0 × 2.5 mm, 5 μm) using ACN:H_2_O 90:10 as eluent at 1.0 mL/min coupled with a Bruker Daltonics MicroTOF QII spectrometer with electrospray detector operating at negative mode.

### 4.2 Plant Material

Seeds of P. macrocarpa were collected by Prof. Dr. Maria Claudia M. Young at *Instituto de Botânica de São Paulo* (23°38′34.02″S and 46°37′17.68″W) in June 2017. A voucher specimen was compared with that previously deposited in the herbarium of IBT SMA, São Paulo, Brazil, under reference SP76791.

### 4.3 Extraction and Isolation

Fresh seeds of P. macrocarpa were dried and milled. The obtained plant material (600 g) was sequentially extracted with hexanes (6 × 1 L) and CH_2_Cl_2_ (6 × 1 L) at room temperature. The solvents were then evaporated under reduced pressure to give hexane (16 g) and CH_2_Cl_2_ (32 g) extracts. As the CH_2_Cl_2_ extract showed activity against the trypomastigote and amastigote forms of T. cruzi (100% parasite death at 300 μg/mL), a portion of this material (30 g) was chromatographed over SiO_2_ eluted with increasing amounts of EtOAc in hexane to afford four groups (A–D). Bioactive group C (3013 mg) was chromatographed over SiO_2_ eluted with hexane:EtOAc (9:1, 8:2, and 7:3) to yield eight groups (C1–C8). Groups C4 (871 mg) and C7 (144 mg) showed activity against T. cruzi (100% parasite death at 300 μg/mL). As previously reported,^6^ group C4 was composed of a mixture of acetylene acetogenins, whereas group C7 was composed of a mixture of **1**–**10**.

### 4.4 Ethics Statement

Test animals used in the experiments were obtained from the animal breeder at the Adolfo Lutz Institute, located in the State of São Paulo, Brazil. These animals were maintained in a controlled environment, housed in sterilized boxes, and given ad libitum food and access to water. BALB/c mice were used to maintain T. cruzi infection and to collect peritoneal macrophages for intracellular assays.^24^ All experimental procedures were approved by the research ethics committee, as indicated in the CEUA-IAL 05/2018 project, in accordance with the guidelines of the Guide for the Care and Use of Laboratory Animals established by the National Academy of Sciences.

### 4.5 Parasite Maintenance

Trypomastigote forms (strain Y) of T. cruzi were obtained from Swiss mice (Mus musculus) previously infected intraperitoneally. The trypomastigote forms were used to infect LLC-MK2 cell cultures consisting of Macaca mulatta kidney epithelial cells (ATCC CCL 7) grown in RPMI-1640 medium containing 2% fetal bovine serum (FBS). The infected cell cultures were maintained in an incubator at 37 °C with 5% CO_2_, as previously described.^25,26^

### 4.6 In Vitro Determination of 50% Effective Concentrations (EC_50_) against Trypomastigote Forms of *T. cruzi*

To evaluate the 50% effective concentration (EC_50_) against the trypomastigote forms of T. cruzi, mixture **1**–**10** was serially solubilized in the RPMI-1640 culture medium in 96-well plates. Subsequently, the trypomastigote forms of T. cruzi were added, at a concentration of 1 × 10^6^ trypomastigotes per well. After an incubation period of 24 h at 37 °C with 5% CO_2_, to determine the viability of trypomastigotes, 20 μL of 10% Alamar Blue (resazurin) was added, followed by a new incubation of the plates for 20 h under the same conditions. At the end of the tests, the plates were read by absorption using a microplate spectrophotometer (Filter Max F5 Multi-Mode Microplate Reader) at 570 nm, as previously described.^27^ Benznidazole was used as a positive control, while cells not subjected to treatment were used as a negative control.

### 4.7 In Vitro Determination of 50% Effective Concentrations (EC_50_) against Amastigote Forms of *T. cruzi*

To evaluate the in vitro activity of the mixture of **1**–**10** against amastigote forms of T. cruzi, macrophages obtained from the peritoneal cavity of female Swiss mice were used by washing with RPMI-1640 medium (without phenol red) and supplemented with 10% SFB. After collecting and counting the cells, they were seeded in Nunc brand 16-well plates and incubated for 18 h at 37 °C in an incubator containing 5% CO_2_. Nonadherent cells were then removed by washing with RPMI-1640 medium. Subsequently, the cells were infected with trypomastigotes at a ratio of 10:1 per well during an incubation period of 4 h. The mixture of compounds, previously diluted in series, was added and incubated for 48 h to determine the EC_50_ against amastigotes. After the incubation phase, the glass slides were fixed using MeOH for 5 min and then stained with Giemsa for 7 min. This procedure allowed for observation under a light microscope. The infection index was obtained in 200 macrophages/slide according to the formula: infected macrophages × number of amastigotes/total number of macrophages. Benznidazole was used as a standard drug.^28,29^

### 4.8 Cytotoxicity against Mammalian Cells

The cytotoxicity of the mixture of **1**–**10** was examined in cells of the NCTC lineage (clone 929) at a density of 6 × 10^4^ cells per well. The cells were cultivated in 96-well plates containing different concentrations of the mixture, serially diluted using RPMI 1640 medium supplemented with 10% fetal bovine serum (FBS). Incubation took place over 48 h, in an incubator maintained at 37 °C with 5% CO_2_. The cell viability of NCTC cells (clone 929) was subsequently evaluated using the MTT colorimetric assay.^30^

### 4.9 Binding Parameters to Membrane Models

Deionized H_2_O for all the procedures of this section was gathered from a Barnstead Nanopure system (Barnstead, Dubuqye, IA, USA), with 18.2 MΩ resistivity and 72 mN/m surface pressure at 20 °C. The lipids 1,2-dipalmitoyl-*sn*-glycero-3-phosphoethanolamine (DPPE), 1,2-dioleoyl-*sn*-glycero-3-phosphoethanolamine (DOPE), and 1,2-didocosahexaenoyl-*sn*-glycero-3-phosphoethanolamine (DDPE) were obtained from MilliporeSigma (Burlington, MA, USA). All lipids were solubilized in CHCl_3_ (HPLC grade, Laboratoire Mat, Quebec, Canada) to yield 0.1–0.5 mg/mL solutions. Unsaturated lipids were stored under an argon atmosphere, with butylated hydroxytoluene at 5 μg/mL as an antioxidant.^31^ DMSO was obtained by MilliporeSigma. The mixture of **1–10** was solubilized in DMSO to yield a 0.5 mg/mL solution.

### 4.10 Surface Pressure Measurements

Surface pressure was determined by the Wilhelmy method in a DeltaPi4 microtensiometer (Kibron Inc., Helsink, Finland) with a 1000 μL Teflon through tube (18 mm diameter and 5 mm depth). Humidity was controlled by a plexiglass box, and temperature was kept at 20 ± 1 °C. 1000 μL of PBS buffer at pH 7.4 was used as the subphase in all experiments. Determination of the saturating concentration of mucins and the mixture was executed by injection of increasing volumes (1 μL) into the subphase and waiting for the equilibrium pressure to be reached. The saturating concentration was the one at which the equilibrium pressure stopped rising. For the mucins, it was determined to be 6 μg/mL (stock solution was 0.5 mg/mL), at a 29.4 mN/m surface pressure; for the mixture, it was 3 μg/mL, at a 45.5 mN/m pressure. These concentrations were used for all of the following experiments. For the lipid monolayer formation, lipids were spread drop by drop to the surface of the subphase, followed by solvent evaporation and waiting for the equilibrium surface pressure to be reached. That would be the initial pressure (Πi) for the plots. Afterward, for the experiments with mucins, the mucin solution was injected into the subphase for a saturating concentration of 6 μg/mL. The surface pressure would rise until an equilibrium pressure (Πe). Surface pressure variation (ΔΠ) was defined as the difference between Πe and Πi. For the experiments with the mixture of **1–10** and mucins, the mixture solution would be injected into the subphase after the equilibrium of mucins (Πe) was reached, at the saturating concentration of 3 μg/mL. Equilibrium was reached to give a new equilibrium pressure (Πe_2_). For these experiments, ΔΠ = Πe_2_–Πi. For the experiments with the mixture of compounds and lipids, without mucins, the initial surface pressure Πi would be obtained as described, and the solution of the mixture would be injected into the subphase for a saturating concentration of 3 μg/mL, and the equilibrium surface pressure (Πe) would be measured afterward.

### 4.11 Binding Parameter Determination

The determination of the binding parameters has been extensively described.^9,22,32^ Briefly, the variation of surface pressure ΔΠ was plotted against Πi and fitted by a linear regression. The intersection of the curve with the *x*-axis results in the maximum insertion pressure (MIP). The uncertainty was calculated according to the covariance of the data. Synergy is defined as 1 + slope, and its uncertainty is obtained by the expression: [σ­(Πe) (1–*r* ^2^)^1/2^]/[σ­(Πi) (*n*–2)^1/2^], where σ is the standard deviation, *r* is the correlation coefficient, and *n* is the number of points. These calculations were made by an online available software (http://www.crchudequebec.ulaval.ca/BindingParametersCalculator).

## Supplementary Material



## Notes

^1^H NMR spectrum (δ, CDCl_3_, 500 MHz) of compounds **1–10**; ^13^C NMR spectrum (δ, CDCl_3_, 125 MHz) of compounds **1–10**; HPLC chromatogram of the mixture of **1–10** from seeds of P. macrocarpa (ACN:H_2_O 9:1 and detection at 210 nm); ESI-HRMS (negative mode) of compound **1**; ESI-HRMS (negative mode) of compound **2**; ESI-HRMS (negative mode) of compound **3**; ESI-HRMS (negative mode) of compound **4**; ESI-HRMS (negative mode) of compound **5**; ESI-HRMS (negative mode) of compound **6**; ESI-HRMS (negative mode) of compound **7**; ESI-HRMS (negative mode) of compound **8**; ESI-HRMS (negative mode) of compound **9**; ESI-HRMS (negative mode) of compound **10**; ESI-HRMS/MS (negative mode) of compound **1**; ESI-HRMS/MS (negative mode) of compound **2**; ESI-HRMS/MS (negative mode) of compound **3**; ESI-HRMS/MS (negative mode) of compound **4**; ESI-HRMS/MS (negative mode) of compound **5**; ESI-HRMS/MS (negative mode) of compound **6**; ESI-HRMS/MS (negative mode) of compound **7**; ESI-HRMS/MS (negative mode) of compound **8**; ESI-HRMS/MS (negative mode) of compound **9**; and ESI-HRMS/MS (negative mode) of compound **10** (PDF)
