# Supplementary material for: Anti-Trypanosoma cruzi Effect of Fatty Acids from Porcelia macrocarpa Is Related to Interactions of Cell Membranes at Different Microdomains as Assessed Using Langmuir Monolayers
Source: ACS Omega. 2025 May 21;10(21):21747–54. doi: 10.1021/acsomega.5c01382 (PMC12138661; doi:10.1021/acsomega.5c01382)
Supplement: Supplementary file 1 [file ao5c01382_si_001.pdf]

The anti-*Trypanosoma cruzi* effect of fatty acids from *Porcelia macrocarpa* is related to interactions of cell membranes at different microdomains as assessed using Langmuir monolayers

SUPPORTING INFORMATION

Ivanildo A. Brito [a], Matheus E. Rosa [b], Elodie Boisselier [c], Vanessa Albuquerque [d], Andre G. Tempone [d], Luciano Caseli [b,*], João Henrique G. Lago [a,*]

[a] Federal University of ABC, Center for Natural and Human Sciences, 09210-180, Santo Andre, SP, Brazil.

[b] Federal University of São Paulo, Department of Chemistry, 09972-270, Diadema, SP, Brazil.

[c] Faculty of Medicine, Université Laval, G1V 0A6, Quebec City, Quebec, Canada.

[d] Butantan Institute, Physiopathology Laboratory, 05503-900, São Paulo, SP, Brazil.

E-mails: lcaseli@unifesp.br (L.C.) and joao.lago@ufabc.edu.br (J.H.G.L.)

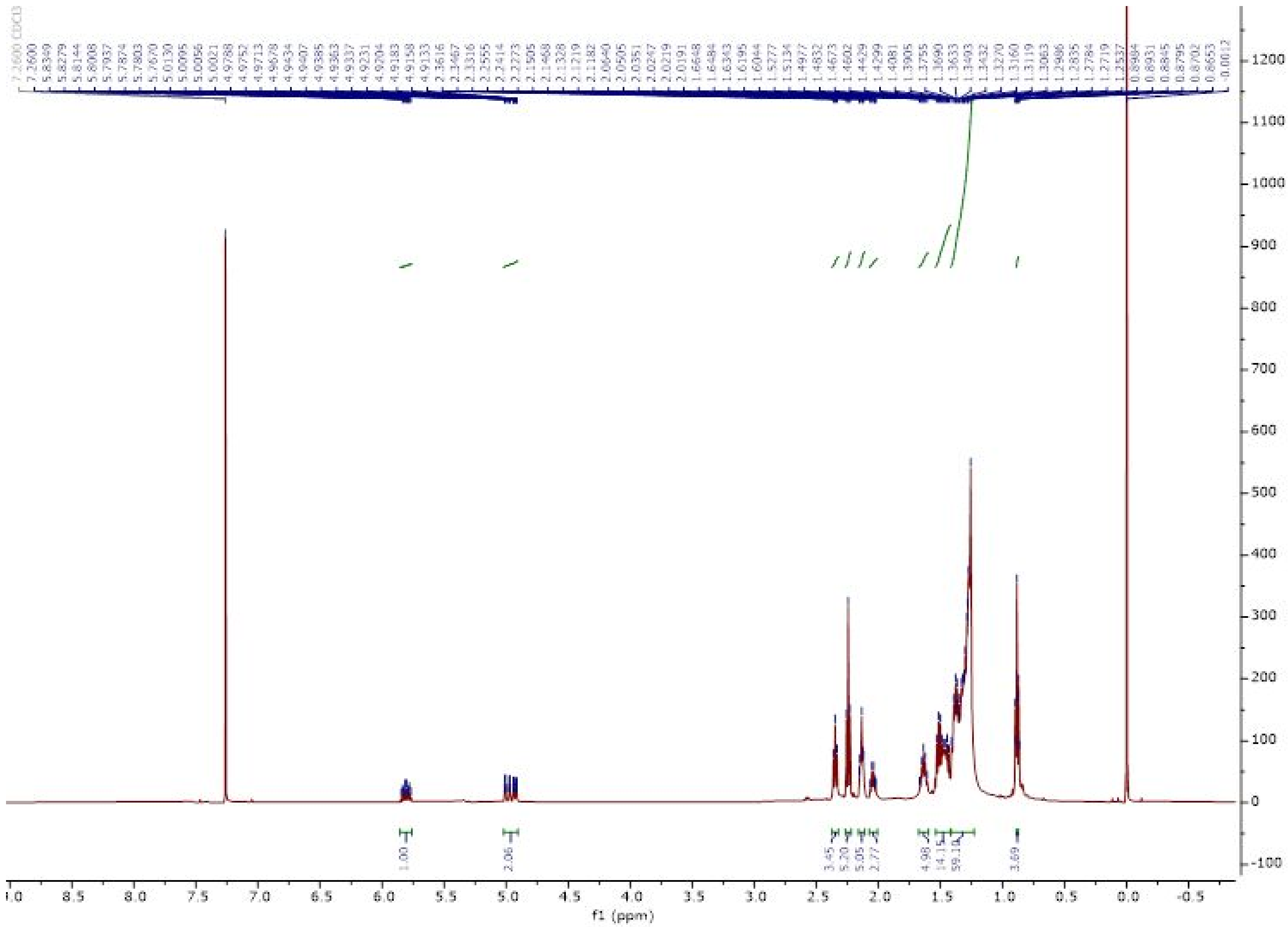

Figure S1 - ^{1}H NMR spectrum (δ, $CDCl_3$, 500 MHz) of compounds **1 - 10**

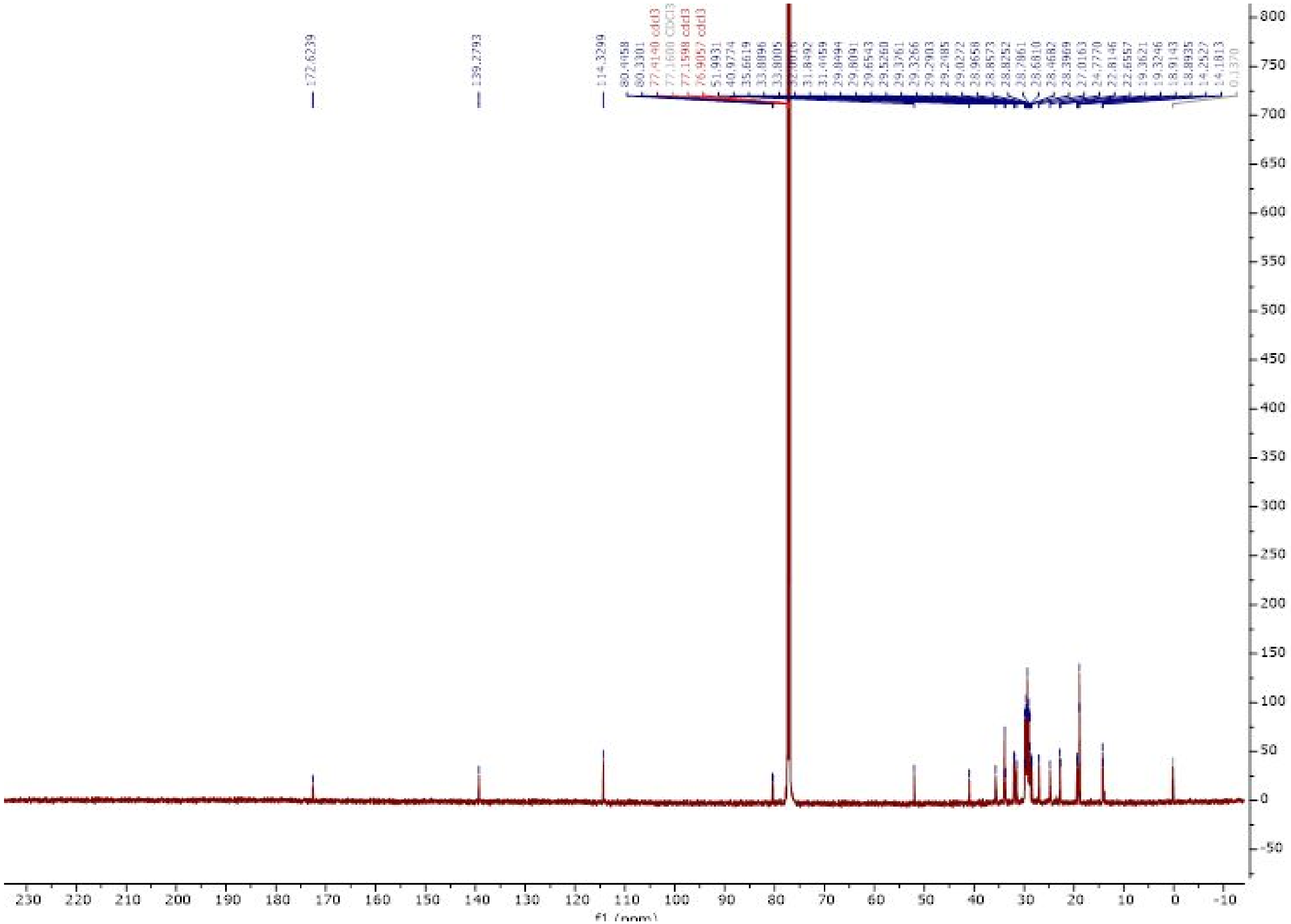

Figure S1 - ^{13}C NMR spectrum (δ, $CDCl_3$, 125 MHz) of compounds **1 – 10**

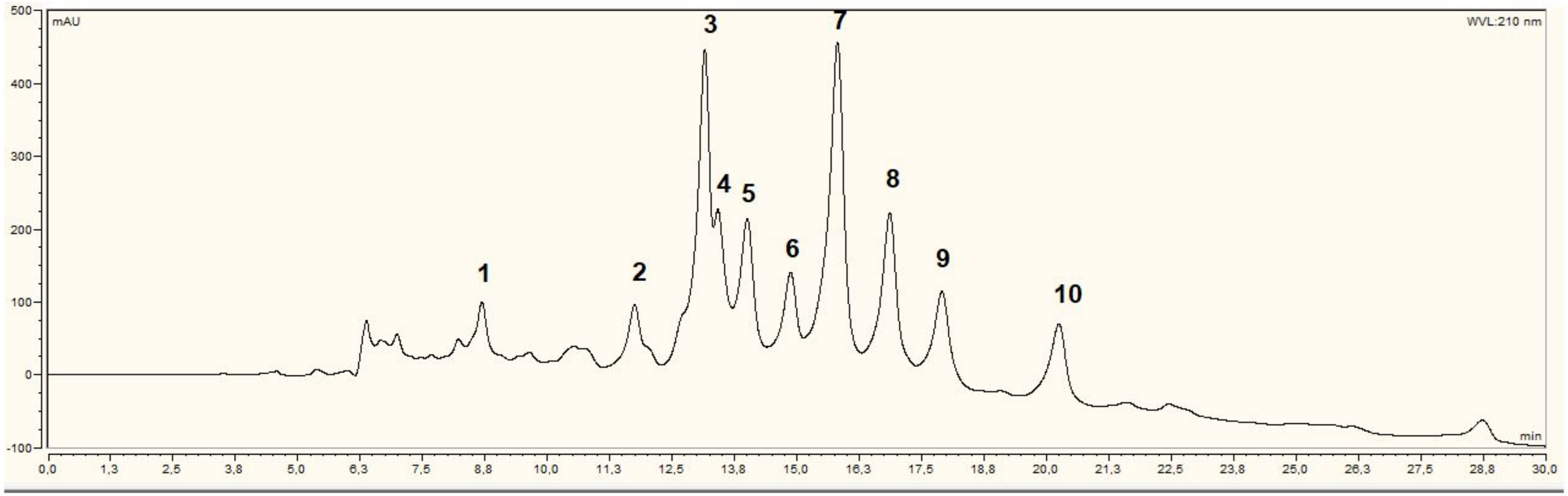

Figure S3 – HPLC chromatogram of mixture of **1 – 10** from seeds of P. macrocarpa (ACN:H_2O 9:1 and detection at 210 nm)

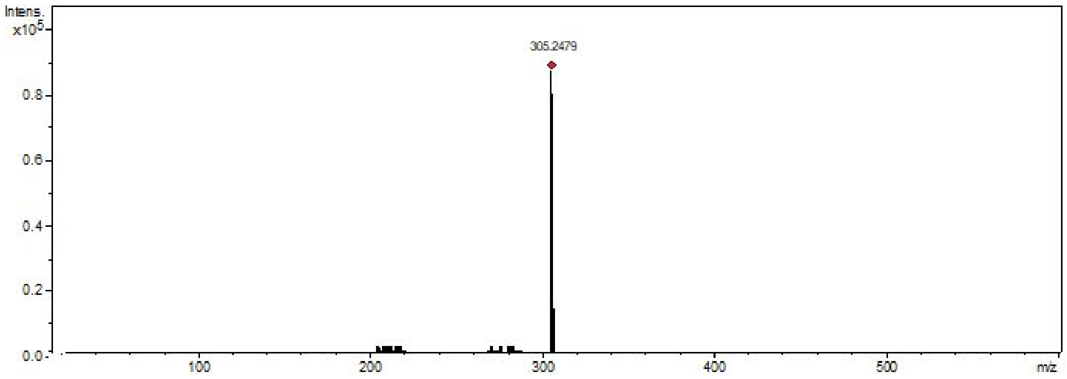

Figure S4 - ESI-HRMS (negative mode) of compound **1**

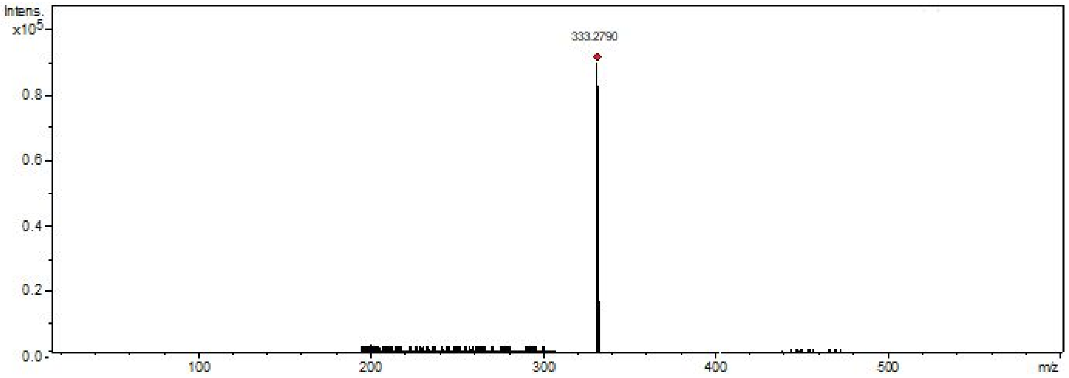

Figure S5 - ESI-HRMS (negative mode) of compound **2**

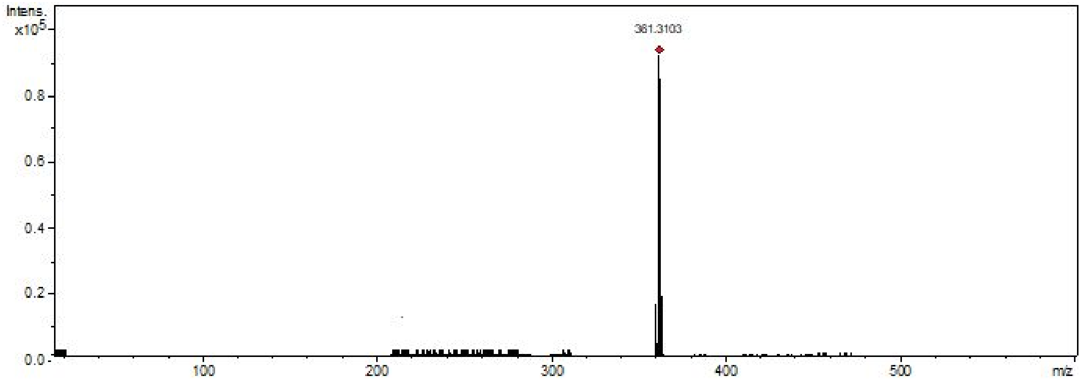

Figure S6 - ESI-HRMS (negative mode) of compound **3**

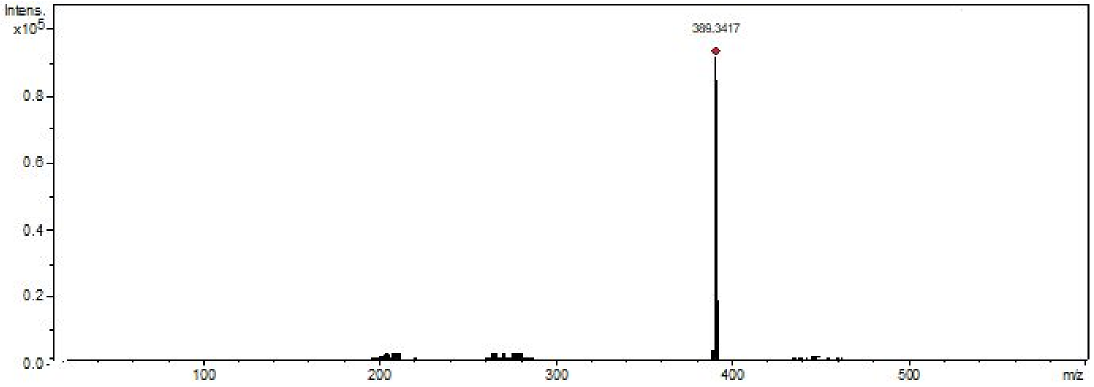

Figure S7 - ESI-HRMS (negative mode) of compound **4**

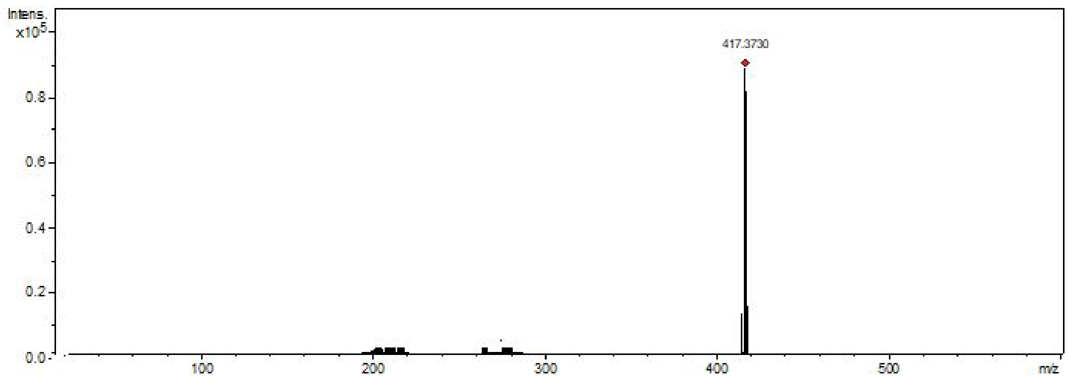

Figure S8 - ESI-HRMS (negative mode) of compound **5**

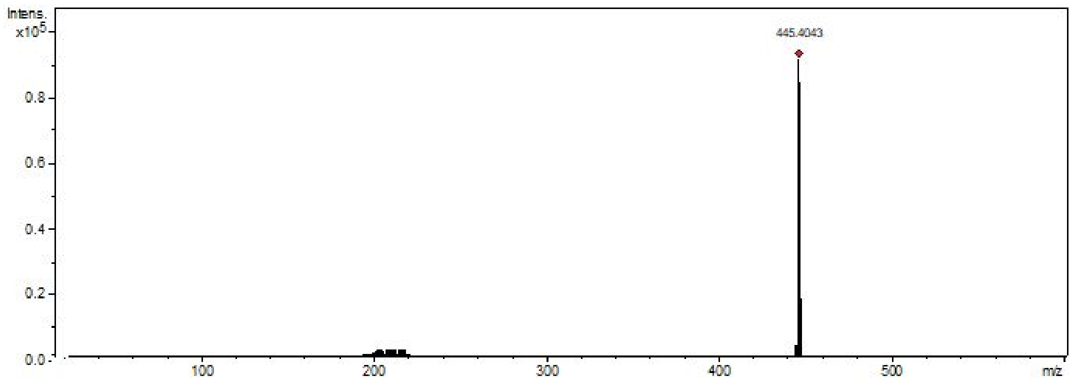

Figure S9 - ESI-HRMS (negative mode) of compound **6**

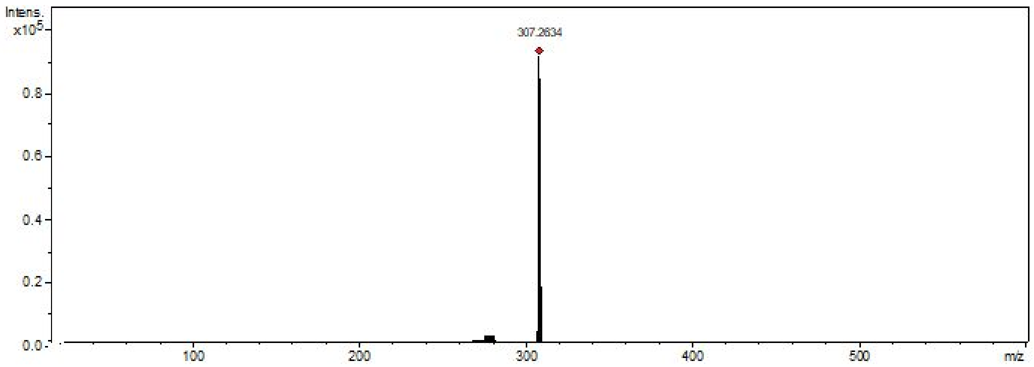

Figure S10 - ESI-HRMS (negative mode) of compound **7**

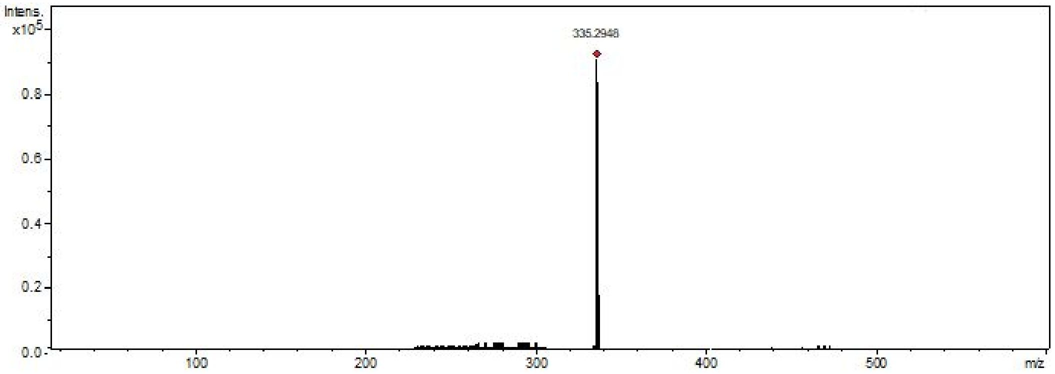

Figure S11 - ESI-HRMS (negative mode) of compound **8**

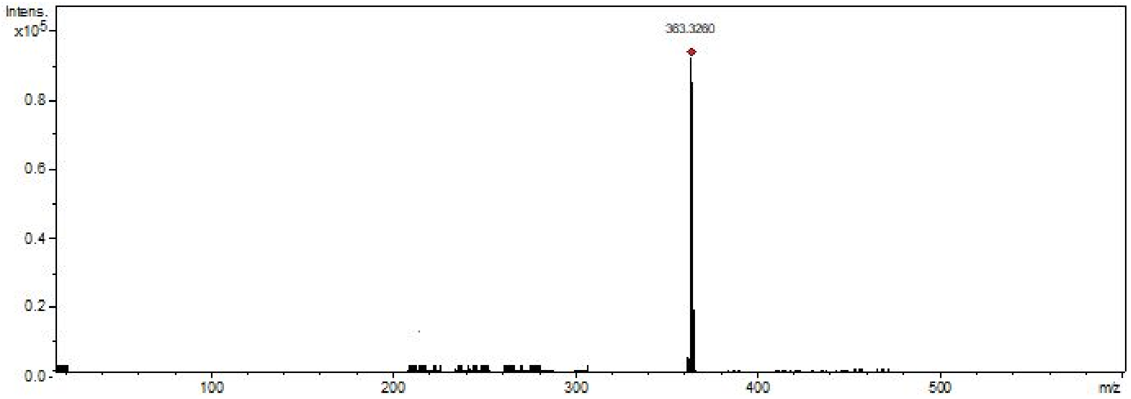

Figure S12 - ESI-HRMS (negative mode) of compound **9**

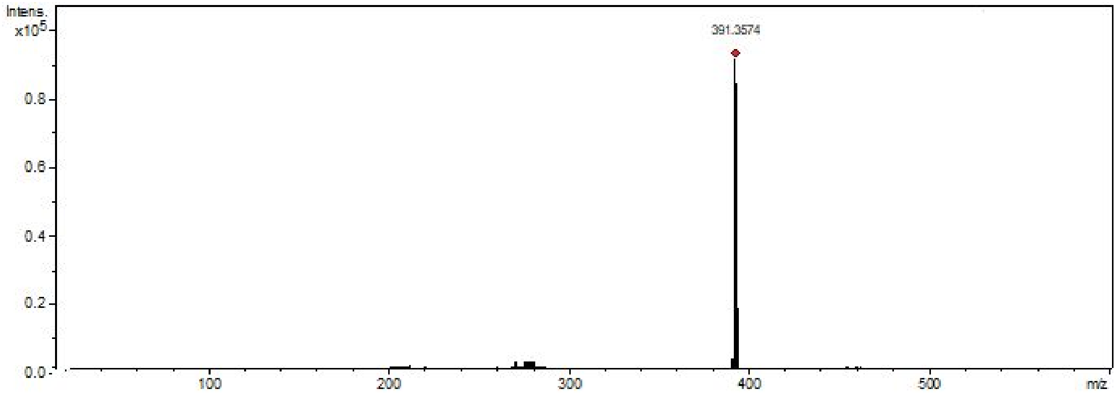

Figure S13 - ESI-HRMS (negative mode) of compound **10**

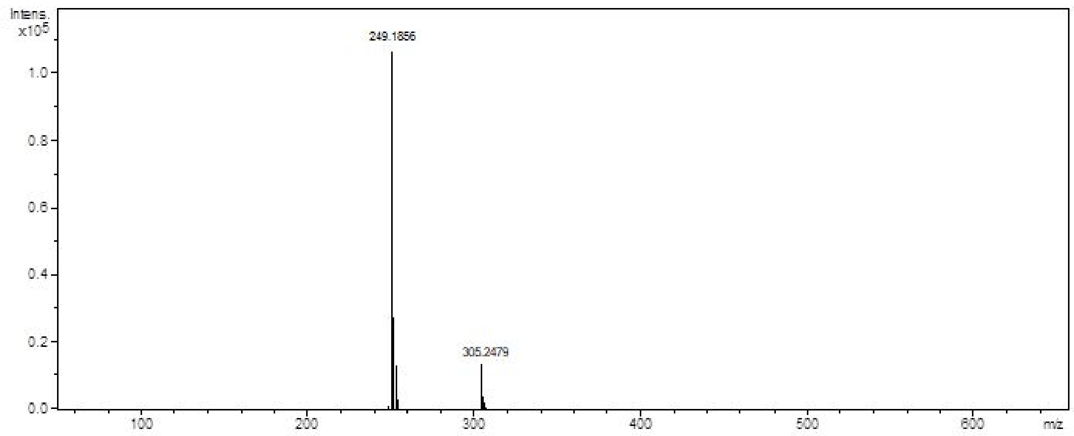

Figure S14 - ESI-HRMS/MS (negative mode) of compound **1**

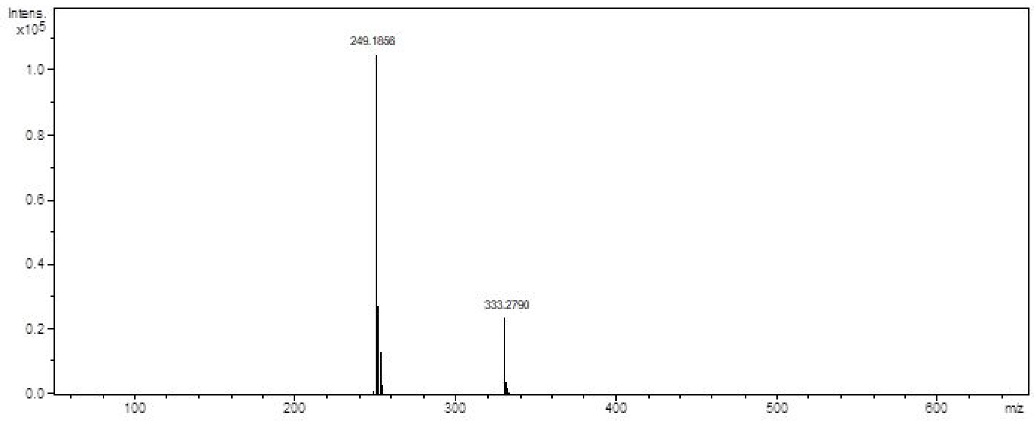

Figure S15 - ESI-HRMS/MS (negative mode) of compound **2**

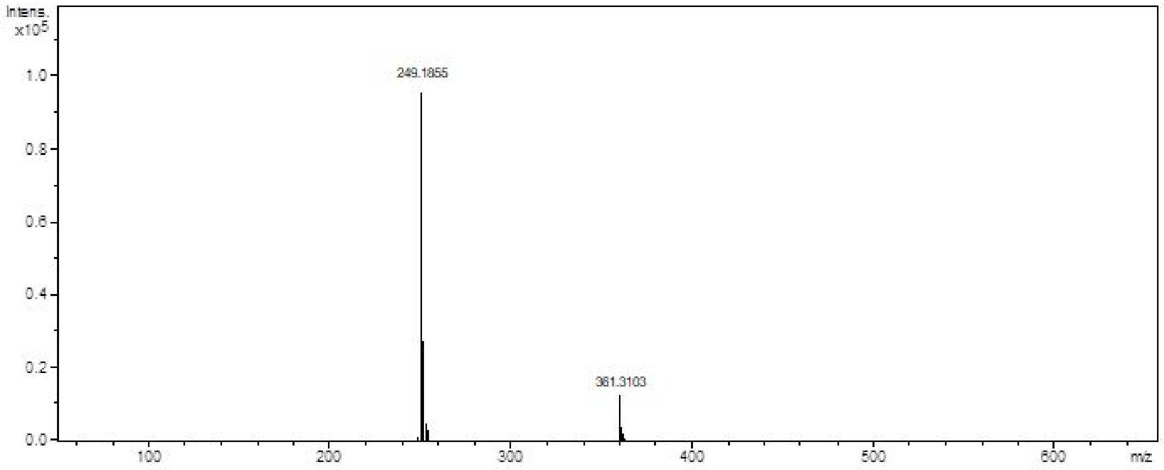

Figure S16 - ESI-HRMS/MS (negative mode) of compound **3**

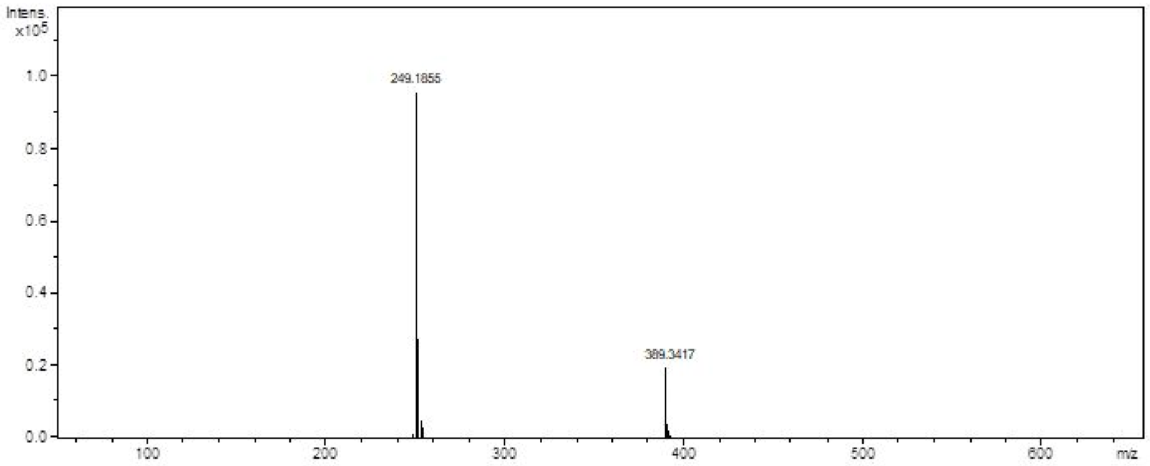

Figure S17 - ESI-HRMS/MS (negative mode) of compound **4**

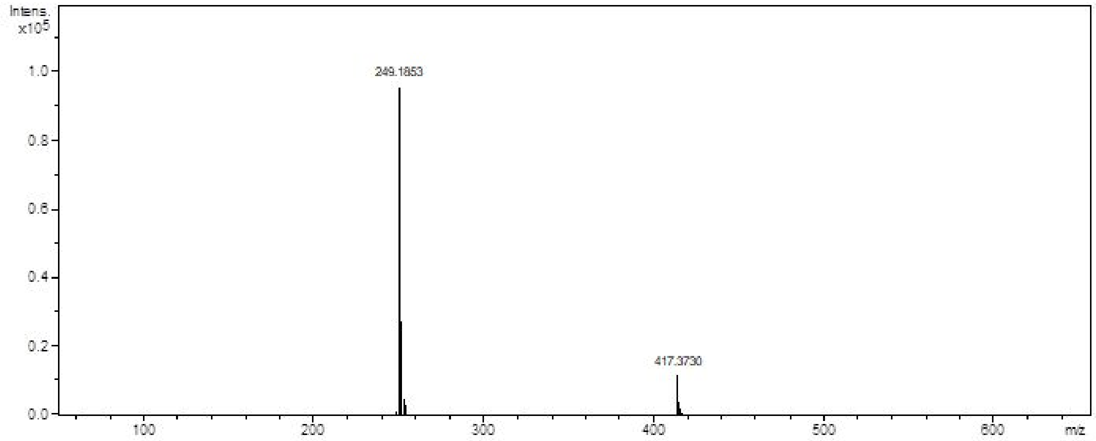

Figure S18 - ESI-HRMS/MS (negative mode) of compound **5**

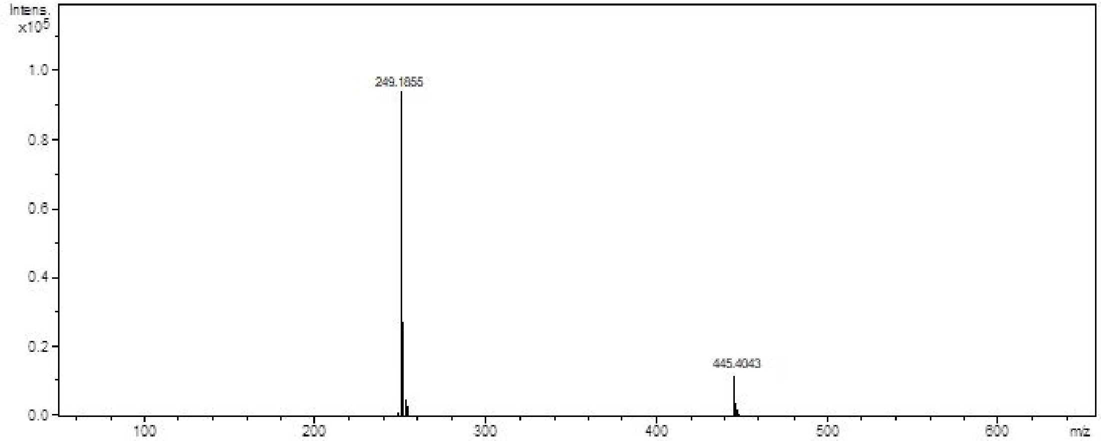

Figure S19 - ESI-HRMS/MS (negative mode) of compound **6**

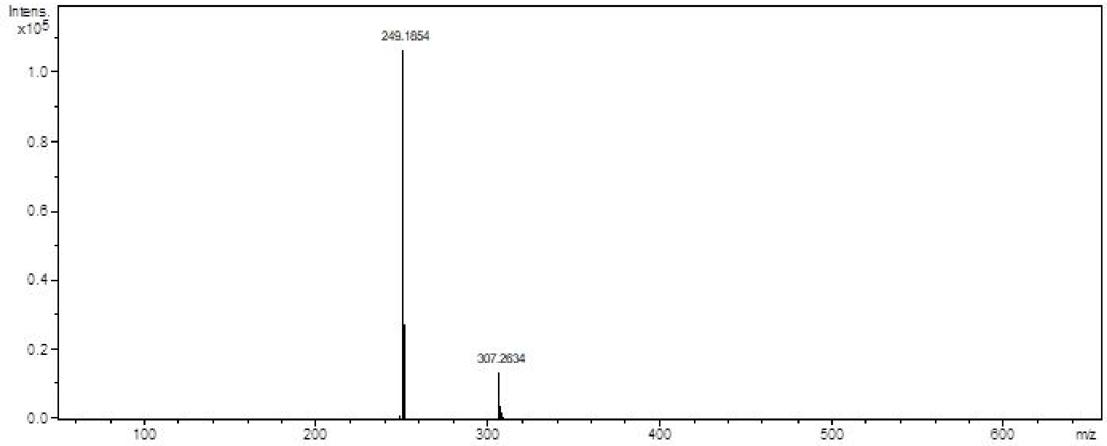

Figure S20 - ESI-HRMS/MS (negative mode) of compound **7**

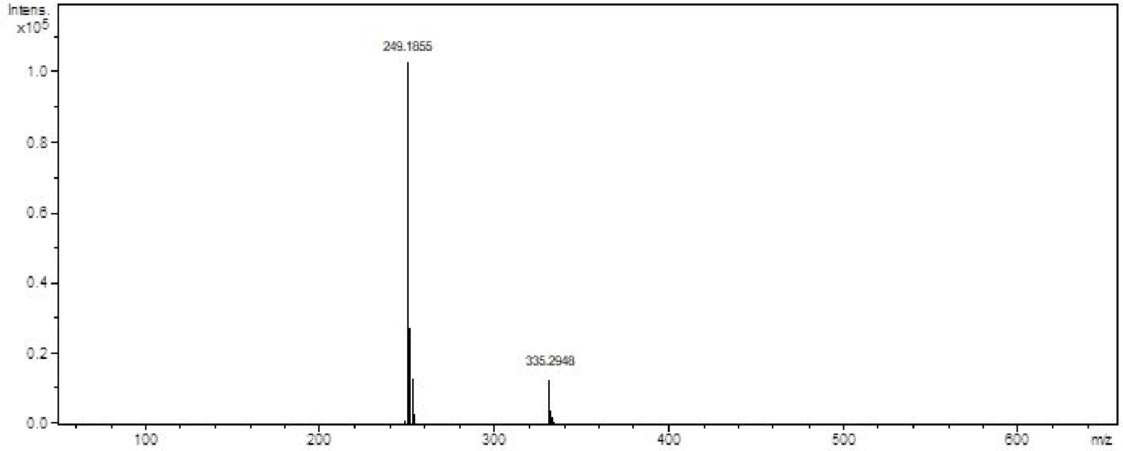

Figure S21 - ESI-HRMS/MS (negative mode) of compound **8**

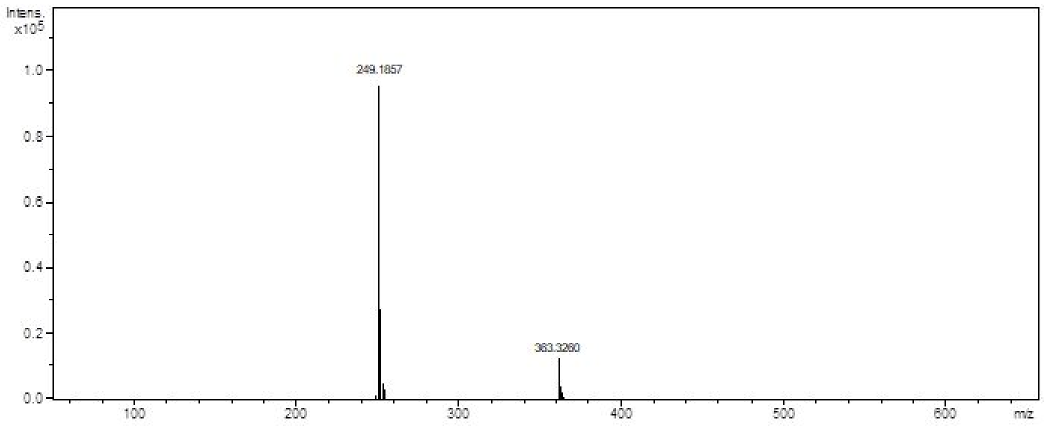

Figure S22 - ESI-HRMS/MS (negative mode) of compound **9**

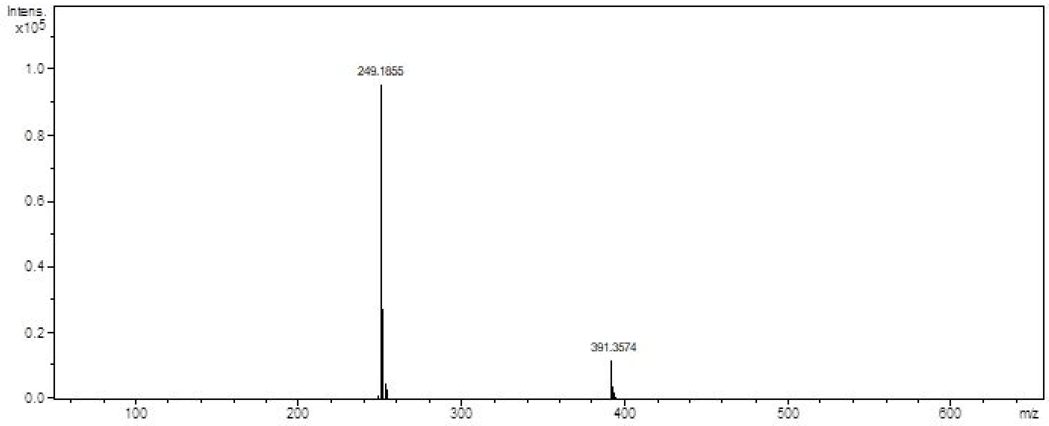

Figure S23 - ESI-HRMS/MS (negative mode) of compound **10**
